# Trait Differentiation within the Fungus-Feeding (Mycophagous) Bacterial Genus *Collimonas*

**DOI:** 10.1371/journal.pone.0157552

**Published:** 2016-06-16

**Authors:** Max-Bernhard Ballhausen, Peter Vandamme, Wietse de Boer

**Affiliations:** 1 Department of Microbial Ecology, Netherlands Institute of Ecology (NIOO-KNAW), Wageningen, the Netherlands; 2 Laboratory for Microbiology, Gent University, Gent, Belgium; 3 Department of Soil Quality, Wageningen University, Wageningen, the Netherlands; University of California-Riverside, UNITED STATES

## Abstract

The genus *Collimonas* consists of facultative, fungus-feeding (mycophagous) bacteria. To date, 3 species (*C*. *fungivorans*, *C*. *pratensis and C*. *arenae*) have been described and over 100 strains have been isolated from different habitats. Functional traits of *Collimonas* bacteria that are potentially involved in interactions with soil fungi mostly negatively (fungal inhibition e.g.), but also positively (mineral weathering e.g.), affect fungal fitness. We hypothesized that variation in such traits between *Collimonas* strains leads to different mycophagous bacterial feeding patterns. We investigated a) whether phylogenetically closely related *Collimonas* strains possess similar traits, b) how far phylogenetic resolution influences the detection of phylogenetic signal (possession of similar traits by related strains) and c) if there is a pattern of co-occurrence among the studied traits. We measured genetically encoded (*nifH* genes, antifungal collimomycin gene cluster e.g.) as well as phenotypically expressed traits (chitinase- and siderophore production, fungal inhibition and others) and related those to a high-resolution phylogeny (MLSA), constructed by sequencing the housekeeping genes *gyrB* and *rpoB* and concatenating those with partial 16S rDNA sequences. Additionally, high-resolution and 16S rDNA derived phylogenies were compared. We show that MLSA is superior to 16SrDNA phylogeny when analyzing trait distribution and relating it to phylogeny at fine taxonomic resolution (a single bacterial genus). We observe that several traits involved in the interaction of collimonads and their host fungus (fungal inhibition e.g.) carry phylogenetic signal. Furthermore, we compare *Collimonas* trait possession with sister genera like *Herbaspirillum* and *Janthinobacterium*.

## Introduction

The limited availability of energy resources is probably the main driver of bacterial diversification in soil [1]. Through constant adaptation, the competition for nutrients has led to the evolution of high bacterial and fungal diversity, sometimes forcing species to interact in order to efficiently acquire carbon [2]. Looking at fungal-bacterial interactions in soil, the bacterial genus *Collimonas* is of particular interest because it evolved the ability to feed on fungi. Collimonads are able to colonize fungal hyphae and to exploit them as their sole source of carbon and energy. This specialized strategy of nutrient acquisition has been termed “mycophagy” [3]. Antifungal traits, such as the production of fungal growth-inhibiting secondary metabolites, are responsible for the destabilization and destruction of fungal cell walls and are therefore thought to play an important role in fungal nutrient acquisition [3, 4]. On the one hand it has been demonstrated that *Collimonas* species differ in the ability to inhibit fungal growth [5]. On the other hand fungi have been shown to vary in sensitivity to attacks by collimonads. Introduction of *Collimonas* strains in a soil with low abundance of indigenous collimonads resulted for example in shifts in fungal community composition without significantly reducing general fungal abundances [6]. This indicates that at least some collimonads have feeding preferences for distinct fungal species. Recently, generalist and specialist feeding strategies have been observed for a variety of other mycophagous bacteria [7]. Next to traits that negatively influence fungi, some collimonads have been shown to carry out functions like mineral weathering that can provide indirect benefits for the fungal host. In the weathering process, *Collimonas* bacteria make inorganically bound minerals available which could not only be of benefit for themselves but also for the fungal hosts. The presence of *Collimonas* on fungal mycelia can also result in increased hyphal branching which might have beneficial effects for the fungus [8, 9].

So far, all studied *Collimonas* strains appear to have mycophagous abilities but none of the strains obligatory depends on fungal nutrition. Differences in fungal inhibition between *Collimonas* strains [5] do however suggest intra-genus diversification, possibly indicating divergence in the relationships with fungi, e.g. specialization for feeding on certain fungal groups.

In this study, we elaborate on the diversification within the genus *Collimonas* using a “trait-based approach” in order to understand how different *Collimonas* strains interact with their fungal hosts. Trait based approaches try to capture the functional capabilities of taxa by measuring a set of characteristics that the organisms possess or are able to carry out [10]. Such approaches have been used in plant ecology for a long time and have not only been used to group organisms in functional categories but also to assess their contributions to the provision of ecosystem services [11]. The interest in functional traits as a tool to study diversification in microbial taxa has become popular among microbial ecologists as well. Most trait-based studies in microbial ecology did however only investigate trait dispersal among higher taxonomic ranks [12].

The aim of this study was to find out if high-resolution phylogenetic grouping of collimonads is coinciding with fine-scale trait distribution. To accomplish this, we first conducted a Multi Locus Sequence Analysis (MLSA), based on concatenated partial sequences of the small subunit of the bacterial ribosome (16S rDNA), DNA gyrase subunit B (*gyrB*) and RNA polymerase β-subunit (*rpoB*). In a second step we related trait investment (the possession or (for quantitative traits) the strength of a trait) to phylogeny by measuring the phylogenetic signal and testing it against a random trait evolution model. We used different methods, relying on gene presence/absence as well as on physiological assays to collect trait data since for some traits, like fungal inhibition or mycophagy, the underlying genes are not or only partly known.

## Material and Methods

### Collimonas strains

We included *Collimonas* isolates from France, Finland, and the Netherlands in this study. The habitats from which the bacteria have been isolated are described in S1 Table. Bacteria that were isolated during this study (see S1 Table) originate from a beech forest or former agricultural field in “Dennekamp” (52° 01ʹ N; 05° 48ʹ E). Permission for sampling here was given by Machiel Bos manager of environmental organisation Natuurmonumenten ZuidWest Veluwe, the Netherlands. Isolates were assigned to the genus *Collimonas* on basis of sequence analysis of the 16s rDNA gene or its digestion with the restriction enzyme *BstBl*. This enzyme cleaves around position 1000 of the16s rDNA at a site (5′-TTCGAA-3′) that is unique for the genus *Collimonas* and therefore allows identification of *Collimonas* bacteria [3]. Experiments were conducted with a set of 88 different collimonads, except for mycophagy assays, weathering—and collimomycin datasets, which included 35 strains, each. We only tested a subset of the strains for mycophagy because the assay is laborious. Data on weathering and the presence of collimomycin gene clusters were retrieved from [13] and [14], respectively.

### Fungi

The fungi that were used in inhibition and mycophagy assays were acquired from the following sources: *Trichoderma harzianum* CECT 2413 was purchased from the Spanish type culture collection CECT (University of Valencia, Spain), *Mucor hiemalis* Wehmer and *Fusarium culmorum* were originally isolated by de Rooij-Van der Goes, Van de Putten [15] from coastal foredunes in the Netherlands. *Pythium ultimum* P17 (an oomycete) was obtained from WUR Applied Plant Research- (PPO, Lisse, the Netherlands).

*Fusarium oxysporum* (CBS619.87), *Aspergillus niger* N400 (CBS120.49) and *Phoma exigua var exigua* (CBS833.84) were purchased from the Fungal Biodiversity Center (CBS-KNAW, Utrecht, The Netherlands). *Rhizoctonia solani* Ag 2–2 IIIb was isolated by the Institute of Sugar Beet Research (IRS, Bergen op Zoom, the Netherlands). *Fusarium culmorum* was cultured on Synthetic Nutrient Agar (SNA), pH 6.8 (KH_2_PO_4_ 1 gL^-1^, KNO_3_ 1 gL^-1^, MgSO_4_7H_2_O 0.5 gL^-1^, KCl 0.5 gL^-1^, Glucose 0.2 gL^-1^, Sacharose 0.2 gL^-1^ and Agar15 gL^-1^), all other fungi on Potato Dextrose Agar (PDA), pH 6.8 (Potato Dextrose Agar, 9.75 gL^-1^; Agar 3.75 gL^-1^).

### Mycophagy assay

The mycophagy assay was conducted for 35 isolates (S1 Table) and each isolate was tested against two phylogenetically different fungi with different lifestyles (saprotroph and pathogen), namely *R*. *solani* and *M*. *hiemalis*. The mycophagy assay was performed as described in Rudnick, van Veen [7]. Briefly, collimonads were inoculated on a petri dish, containing phytagel medium. Use of phytagel enables to create a semi-solid medium that contains almost no energy resources [16]. In the next step, the fungus was introduced in the middle of the petri dish on a nutrient rich plug that was placed on top of a metal disc thereby avoiding diffusion of nutrients from the plug into the phytagel. While naturally expanding, the fungal hyphae were confronted with the surrounding bacteria. Bacteria increased in biomass if they were able to grow on living fungal tissue or fungal exudates as their sole source of carbon. After incubation, the bacteria were washed off the microcosm and the optical density (OD_600_) of the bacterial suspension was determined and compared with controls (bacteria- or fungus only control). Mycophagy ratios (OD_treatment_/OD_control_) were calculated with the higher of the two controls, averaged over 3 replicates. If a significant positive difference between the fungal treatment and the control was detected the respective bacterium was considered to be mycophagous.

### Swarming and siderophore assay

The swarming assay was done on M9 medium with 0.5% Agar, as described in Xavier, Kim [17], scored after 48 hours of incubation at 20°C, subsequently overlaid with CAS medium [18] and scored for siderophore production after 5 hours. The program ImageJ [19] was used to measure the extension of the typical yellow margin around the colonies indicating siderophore production (average of 5 different spots).

### Fungus inhibition assays

Assays were conducted on Water Yeast Agar (WYA) (KH_2_PO_4_ 1 gL^-1^, NaCl 5 gL^-1^, Yeast extract 0.05 gL^-1^ and Agar 20 gL^-1^, pH 6.8) using standard size petri dishes. *Collimonas* bacteria were pre-cultured on Tryptic Soy Agar (TSA) (KH_2_PO_4_ 0.5 gL^-1^, NaCl 2.5 gL^-1^, Yeast extract 0.05 gL^-1^, Tryptone 1.5 gL^-1^ and Agar 10 gL^-1^, pH 6.8) and inoculated on a 2 cm x 6 cm zone, 4 days before the fungi/oomycete were introduced as a plug from the margin of an actively growing colony. Assays were incubated at 20°C and scored depending on the growth speed of the fungus, after 4 days for *P*. *ultimum*; after 7 days for *R*. *solani*, *M*. *hiemalis*, *F*. *culmorum and T*. *harzianum;* after 14 days for *F*. *oxysporum* and *P*. *exigua*; and after 21 days for *A*. *niger*.

### Chitinase assay

Assays for chitinolytic activity were conducted on colloidal chitin agar (CYA) [20] and scored for halo formation around the colonies after 9 days of incubation at 20°C. Before transfer to CYA, collimonads were pre-grown on TSA.

### Choice of housekeeping genes

Housekeeping genes coding for DNA gyrase subunit B (*gyrB*) and RNA polymerase β-subunit (*rpoB*) were chosen because of their ubiquity in bacteria. They code for essential cell metabolic functions, are conserved and mostly change due to insertions, deletions or point mutations. *RpoB* and *gyrB* are not located adjacent to genes that encode for outer surface proteins or hypothetical proteins and are single copy genes. This is important to consider when designing an MLST scheme, since multiple gene copy numbers and/or locations next to elements that are subjected to higher selective evolutionary pressures might distort phylogenetic grouping of the isolates [21].

### Primer design

Primers for the amplification of the two housekeeping genes *rpoB* & *gyrB* were designed based on the corresponding sequences in the genome of *Collimonas fungivorans* Ter331 and related bacteria that belong to the family *Oxalobacteraceae*. We decided to design primers for the family *Oxalobacteraceae* rather than for the genus *Collimonas* to ensure amplification from all *Collimonas* isolates. The pipeline Primer Prospector [22] was used for primer design & specificity testing. The main criterion for primer selection was that *de novo* primers had to match with *Collimonas fungivorans* Ter331. Parameters were then adjusted to include as many related strains as possible while not having more than 10% degeneracies in the primers. Finally primers were sorted and pairs selected based on similar GC-content and annealing temperatures. Information on *rpoB* & *gyrB* primers used in this study can be found in the S3 Table.

### Culturing, PCR amplification & sequencing

Strains were either grown on 10% TSA plates or in liquid TSB. Single colonies were suspended in 100 μl water, heated 10 min at 98°C and used directly for PCR amplification (ColonyPCR). Alternatively, DNA was isolated using Phenol/Chloroform, as described in [23]. PCR reactions for 16S rDNA were conducted with the primers 27f and 1492r [24]. The 25 μl reactions contained 18.14 μl H2O, 2.5 μl 10x PCR-buffer containing 2 mM MgCl2 (Roche Scientific, Woerden, the Netherlands), 0.2 mM of each dNTP (Roche Scientific, Woerden, the Netherlands) and 0.4 μM of each primer, 1 U Fast Start High Fidelity Polymerase (Roche Scientific, Woerden, the Netherlands) and 1 μl DNA template. Thermal cycling conditions for partial bacterial 16S gene amplification were as follows: pre-denaturation of 10 min at 95°C to break the cells open, initial denaturation of 94°C for 2 min, followed by 34 cycles of 94°C for 30 sec, 55°C for 1 min and 72°C for 90 sec with a 1 sec increment per cycle and a final elongation step at 72°C for 10 min. Nitrogenase (*nifH*) and violacein genes (*VioA* & *VioB*) were amplified as described in [25] and [26], respectively, and fragment size was examined on standard (1.5 w/v) agarose gels. PCR reactions to amplify *rpoB* and *gyrB* genes were carried out in 25 μl reactions, containing: 18.14 μl H_2_O, 2.5 μl 10x PCR-buffer incl. 2mM MgCl_2_ (Roche Scientific, Woerden, The Netherlands), 0.2 mM of each dNTP (Roche Scientific, Woerden, The Netherlands), 0.4 μM of each Primer, 1U Fast Start High Fidelity Polymerase (Roche Scientific, Woerden, The Netherlands) and 1 μl template DNA. Cycling conditions consisted of an initial denaturation of 95°C for 2min, followed by 30 cycles of 95°C for 30 sec, 60°C for 30 sec and 72°C for 40 sec with a final elongation step at 72°C for 7min. PCR products were examined by a standard (1.5 w/v) agarose electrophoresis and cleaned with 20% PEG8000 (SigmaAldrich) before being sent to Macrogen (Amsterdam, The Netherlands) for sequencing (in case of 16s, *rpoB* and *gyrB*). Sequences were uploaded to the European Sequence Archives (ENA), and can be accessed under accession numbers KU311378—KU311465 (16S rDNA), KU311290—KU311377 (gyrB DNA) and KU311202—KU311289 (rpoB DNA).

### Phylogenetic analysis

Quality check and editing of the obtained sequences was done using the software SequenceScanner (Applied Biosystems) and BioEdit [27]. Alignment and curation of sequences was done with the program MEGA 5.2 [28] and ClustalW. Sequences were concatenated with DAMBE 5.3.9 [29] and MEGA 5.2 was used to construct a Neighbor-Joining Tree with standard settings. The pairwise deletion option was used to treat missing data and gaps; finally the tree was tested with 100 bootstrap replicates.

### Phylogenetic signal

Phylogenetic signal describes how well phylogeny predicts the trait distribution on a phylogenetic tree [10].We used different methods to statistically test for the existence of such signal, depending on the nature of the trait data. All methods assume that random trait evolution is best described by a random walk of Brownian motion (BM) along the branches of the phylogenetic tree [10, 30].They give an indication of the strength of the signal and test whether it significantly differs from one derived from random trait distribution. For continuous trait data we used Bloomberg’s K [31] (K = 1 indicates trait evolution following BM, K < 1 less trait divergence than BM and K > 1 clustering of traits which is higher than expected under BM), for discrete traits Pagel’s λ [32] (λ = 0 equals BM trait evolution, λ = 1 equals trait clustering higher than to be expected on basis of BM), and for binary traits Fritz’ and Purvis’ D [30] (D ≤ 0 characterizes a higher trait clumping than to be expected under BM, D = 1 suggests a random trait evolution). Statistically the signal calculation differs, due to the continuous, discrete or binary nature of the trait data. For details on phylogenetic signal calculation of different trait data we refer to the review by Münkemüller, Lavergne [10]. Phylogenetic signal was calculated using the software “R” and the packages “phytools”,” geiger” and “caper”. Since negative or zero branch lengths interfered with the phylogenetic signal calculation, the smallest branch length (in case of no negative branches but zero branch lengths) or the branch length that would equal a negative branch to zero plus 0.00000001 was added to all branches of the tree.

### Statistics

Correlations between traits were calculated using different methods, depending on the nature of the variables. For relationships between continuous variables such as weathering and mycophagy ratios, we used Pearson’s correlation coefficient or linear regression. Relations between continuous and 2 level categorical (binary) data like purple pigmentation (violacein) and mycophagy ratios e.g. were investigated using t-tests. When binary data were tested against binary data, Pearsons chi-squared test was used. This was for example the case for fungal inhibition and purple pigmentation. With categorical data that had more levels, we performed analyses of variance against continuous data. This was for example the case for siderophore production and swarming intensity. After the ANOVA we performed Turkey’s HSD test to assign significance. Categorical data with more levels were tested against one another or against binary categorical data using logistic regression models. This was for example the case for the relationship between different habitat types and swarming intensity. A summary of all calculations can be found in S2 Table. All statistics were done using the program R.

### External data

Collimomycin data were gathered from Fritsche, van den Berg [14], weathering data from Uroz, Calvaruso [13], and habitat data from Uroz, Calvaruso [33], Hoppener-Ogawa, Leveau [34], Mannisto and Haggblom [35] and Nissinen, Mannisto [36].

## Results

### Phylogenetic tree comparison

**Fig 1:**
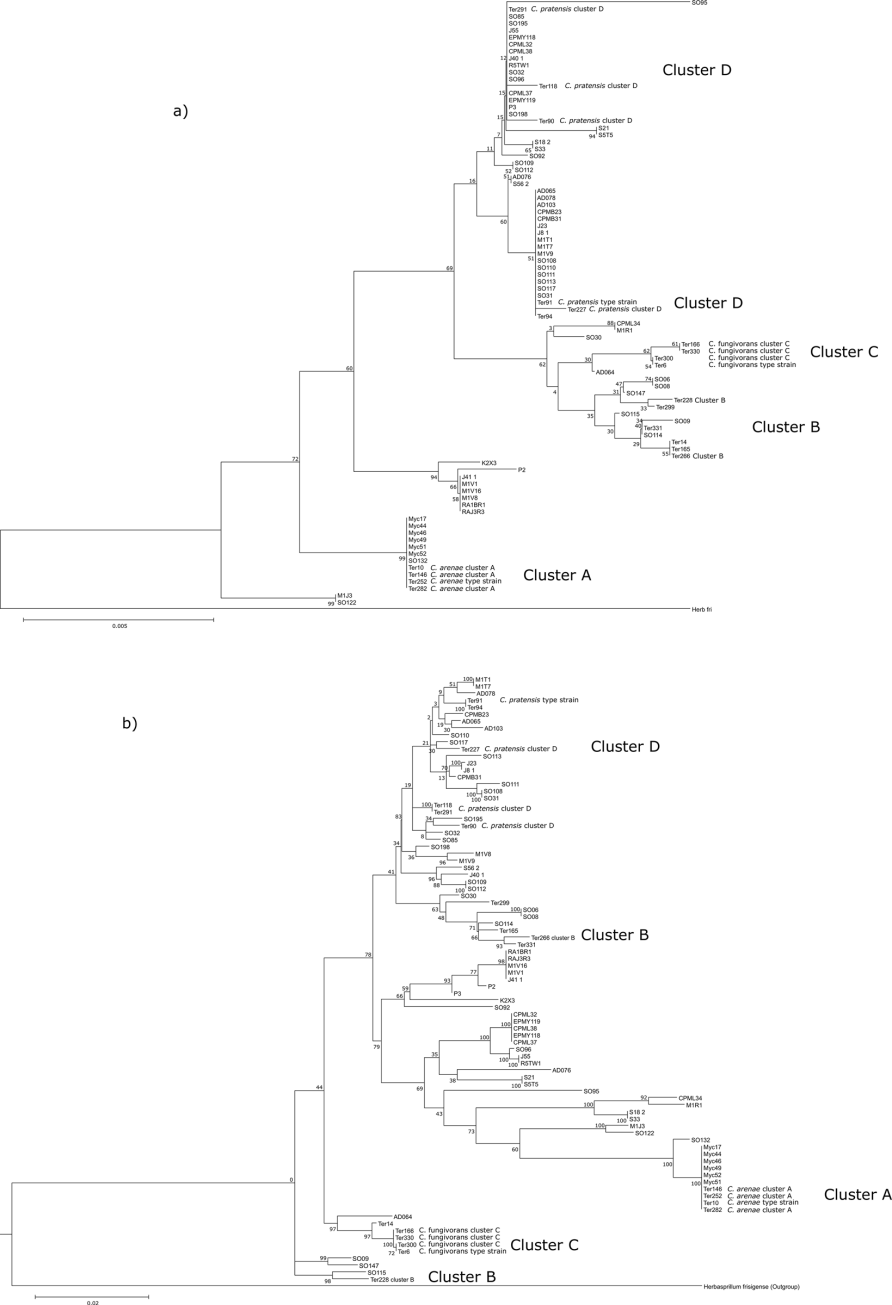
Comparison single gene derived phylogeny a) (16s) and concatenated gene derived phylogeny b) (16s, rpoB, and gyrB) Bacterial strain assignment to clusters as suggested by Hoppener-Ogawa, de Boer [38] is indicated. The trees were tested with 100 bootstraps and the respective support values are indicated at the tree nodes.

Overall, the MLSA tree provides a higher taxonomical resolution than the phylogeny that is based on 16s rDNA sequences only (Fig 1). Still, some *Collimonas* strains could not be differentiated from one another even with the MLSA phylogeny. Such strains have, however, been isolated from very different habitats and/or samples [33–37], thus probably represent different strains. The higher resolution of the MLSA also becomes apparent when comparing it with the grouping of strains previously assigned to already described species *C*. *fungivorans (cluster B and C)*, *C*. *pratensis (cluster D)* and *C*. *arenae (cluster A)* [37, 38]. In the MLSA tree, several differences are apparent: a) Cluster B splits into 2 separate clusters, b) The pre-defined clusters [37] are diverse, especially cluster D seems to harbor strains that form subgroups inside the cluster, c) We find evidence for strains that form clusters which have not been described, yet. The latter strains are RA1BR1, RAJ3R3, M1V16, M1V1, J41_1, P1, P2, K2X3 and SO92 and also the group consisting of CPML32, EPMY119, CPML38, EPMY118, CPML37, SO96, J55, R5TW1, AD076, S21 and S5T5. The two groups split from the branch forming cluster A with reasonable bootstrap support (79 and 69, respectively).

### Mycophagy assay

Average mycophagy ratios varied between isolates and were significantly different between the two test fungi (*M*. *hiemalis*: 64.7, *R*. *solani*: 5.7, P ≤ 0.0001 (t-test)). In the case of *M*. *hiemalis*, 3 out of 36 strains (SO122, AD076, and Ter6) did not show significant mycophagous growth (S1 Table). *R*. *solani* did not significantly support growth of 13 strains (M133, K2X, SO92, J55, Ter118, SO113, Ter331, CPML32, S33, SO132, AD076, Ter6, and S5T5). We decided to exclude such non-significant strains from the subset of bacteria that were further analyzed for mycophagy as those strains could potentially be mycophagous but the variation between replicates was too high to allow conclusions which could bias follow-up data analyses like the calculation of the phylogenetic signal or correlation analyses between traits.

### Swarming assay

**Fig 2:**
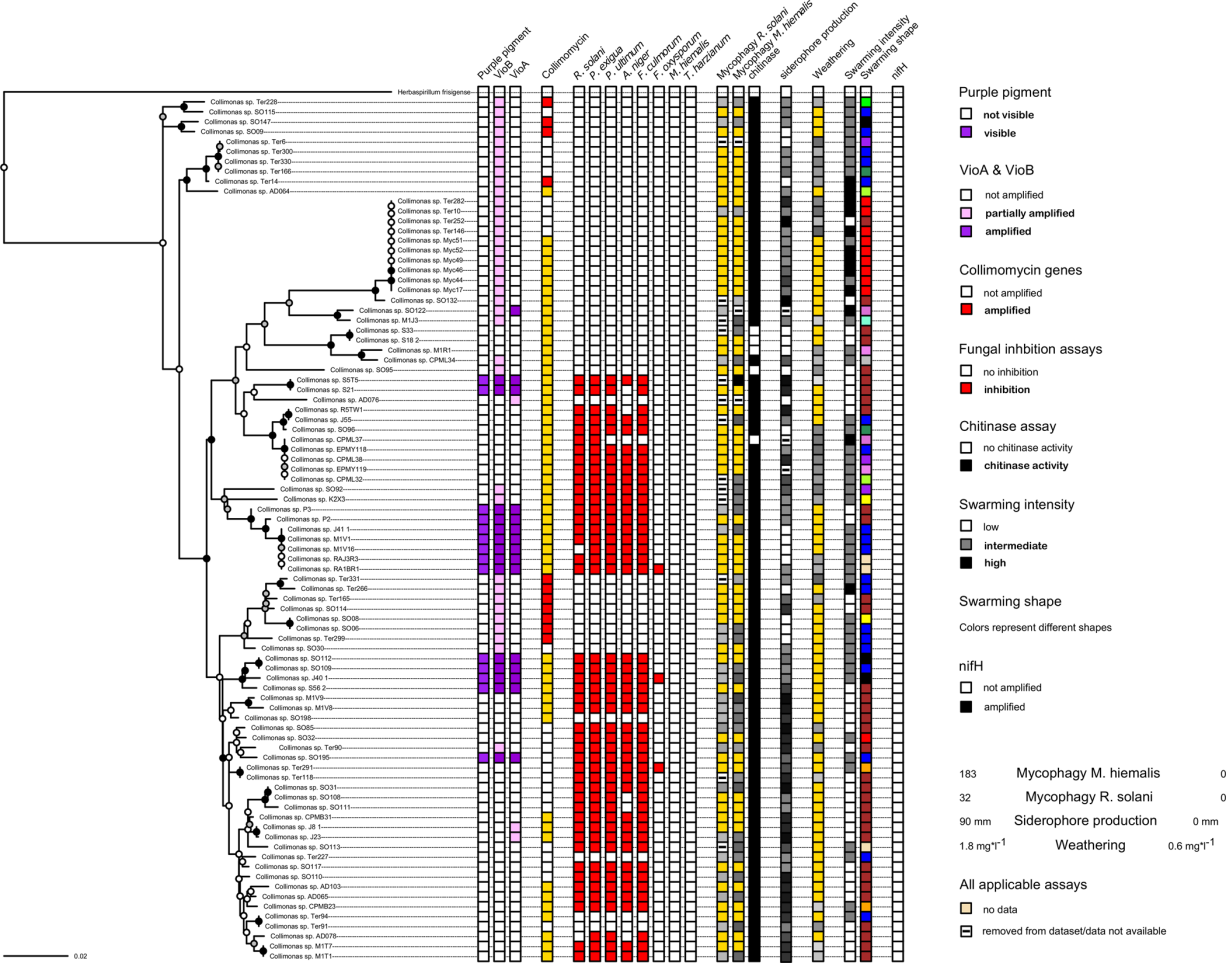
Overview on grouping of collected trait data with the concatenated housekeeping gene phylogeny. The tree was tested with 100 bootstraps, nodes with white circles represent bootstrap support values lower than 50, grey higher than 50 and black higher than 75.

We observed differences in swarming abilities among collimonads. Approximately one third (37 of 90) isolates were not able to swarm at all. The group of swarmers could be divided into slow swarmers which did not colonize the whole petri dish (94 mm dia, 16 mm height) in 48 hours (41 isolates), moderate swarmers which colonized the whole petri dish in 48 hours (12 isolates) and fast swarmers that colonized the whole petri dish in less than 48 hours. Interestingly, collimonads from cluster D (*C*. *pratensis)*, earlier described to produce smaller colonies than other collimonads, were also found to be less capable of swarming. Furthermore, swarming isolates could be grouped into different swarming shapes (for the description of shapes we refer to S1 Table). Similar shapes were given the same color in Fig 2.

### Siderophore assay

Siderophore production, indicated by an orange/yellow circle around the colony margin, varied from 0 to 89 mm in diameter, ranging from no siderophore production to a halo that nearly covered the whole petri dish.

All isolates that were scored siderophore positive, showed orange/yellow halos around the colonies, typically indicating the presence of hydroxamate-type siderophores [18]. The ability to produce siderophores differed between the tested isolates. The majority (70 isolates) produced siderophores at the colony margin and underneath the colony. For 3 isolates, the amount of siderophores was not measureable since the colony already covered the whole plate when the assay was conducted. 10 strains produced siderophores only underneath the colony, 3 isolates only at the center of the colony, and 17 isolates did not show siderophore production at all.

### Inhibition assays

Fungal inhibition was either scored negative (no difference in fungal growth with or without presence of a *Collimonas* strain) or positive (fungal growth was clearly slowed down/stopped by the bacterial strain in comparison to the fungus only control). For the fungi *R*.*solani*, *M*. *hiemalis*, *F*. *oxysporum*, *F*. *culmorum*, *A*. *niger*, *P*. *exigua*, *T*. *harzianum* and the oomycete *P*. *ultimum*, 43, 90, 85, 42, 47, 41, 90, 42 strains had no effect on mycelial extension and 47, 0, 5, 48, 43, 49,0 and 48 strains delayed or stopped mycelial extension, respectively. Generally, we found little variation in the inhibition pattern of the fungi by *Collimonas* bacteria. Most collimonads were either able to inhibit the growth of a whole range of fungi or they were not able to inhibit at all.

### Chitinase assay

All but 5 strains were scored positive for chitinase production on CYA. The five strains showing no clearing of colloidal chitin were SO95 (related to cluster A), CPML(37) (part of a newly formed cluster), M1R1, S33 and S18_2 (all three related to cluster A).

### Genetic analyses

All tested strains scored negative for the presence of nifH, a key gene involved in nitrogen fixation. Production of violacein could be confirmed by detection of PCR amplicons of *VioA* & *VioB* gene fragments (typical sizes being about 1000bp and 900bp, respectively (Hakvåg, et al. 2009)) in the purple colored isolates SO195, SO109, P2, P3, SO112, S5T5, RA1BR1, RAJ3R3, M1U16, J41_1, J40_1, S21, M1U1 and S56_2. Wrong size PCR amplicons (not sequenced) were obtained from strains AD076, J8_1 and J23 with *VioA* primers and in strains SO06, SO08, SO09, SO95, Ter14, Ter266, Ter299, Ter6, SO30, Ter330, Ter331, AD064, Ter300, K2X3, Myc51, Ter146, Ter228, SO147, Myc49, Ter10, Myc52, M1J3, SO115, Myc17, Ter166, Myc46, Ter165, CPML34, SO92, Myc44, SO114, Ter90, Ter282, Ter252, SO132 and SO122 with *VioB* primers.

In S1 Table, an overview is given of phylogenetic-, functional -, and habitat-related information gathered for all strains. For an overview on taxa and associated traits, all trait data are plotted next to the MLSA phylogeny in Fig 2.

### Correlations between traits

**Fig 3:**
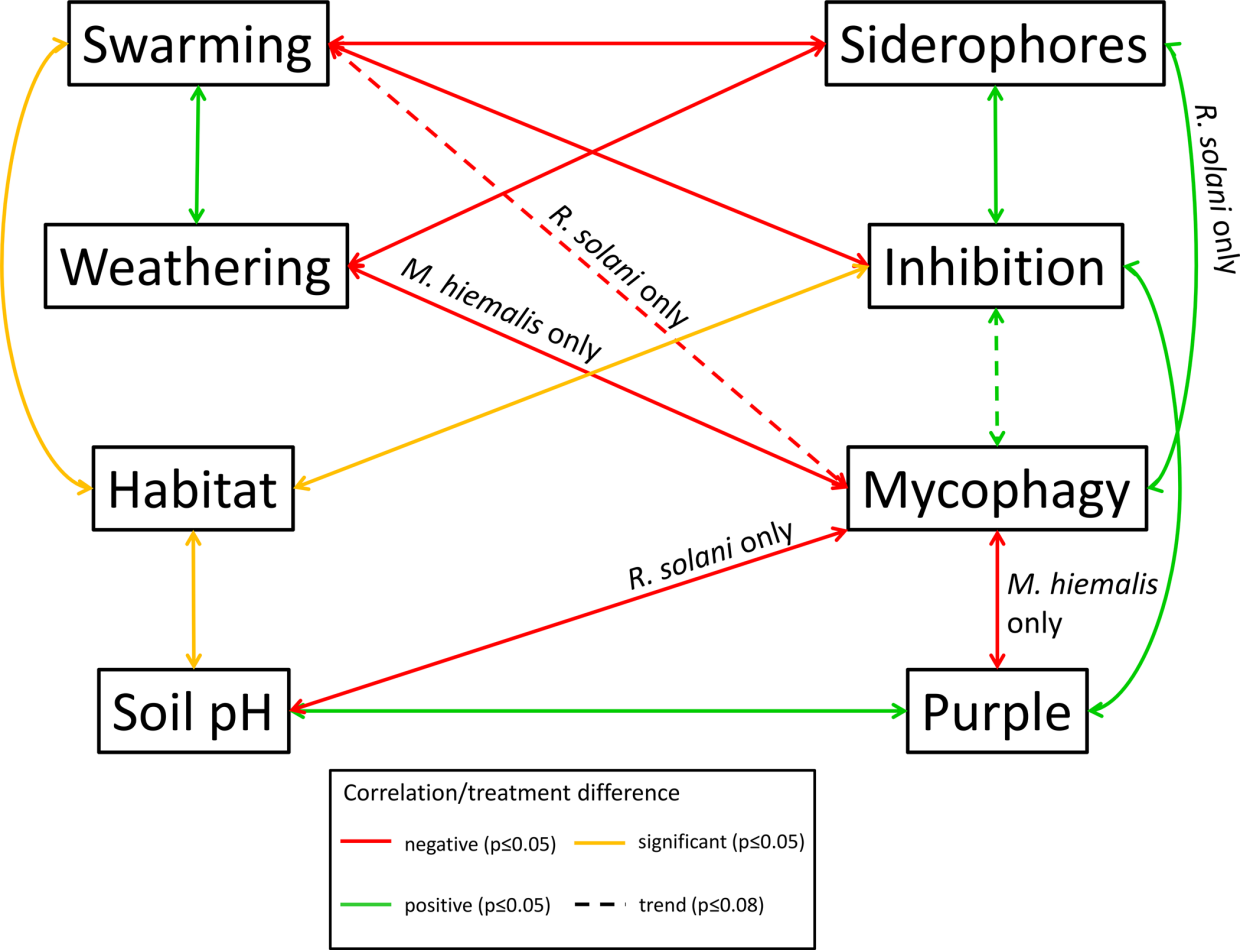
Graphical summary of trait correlations. Significant (p ≤ 0.05) negative correlations are indicated in red, positive correlations in green, correlations close to significance (p ≤ 0.08) are indicated with dashed lines. Significant differences between treatments of categorical variables are indicated in yellow. Indication of *R*. *solani* only *and M*. *hiemalis* only implies that the correlation of a trait with mycophagy is only for mycophagous growth of collimonads on either of these fungi.

We found that of the studied traits several were significantly correlated (Fig 3). Significant positive correlation (p ≤ 0.05) was found between swarming and weathering, between siderophore production and fungal inhibition (all fungi except for *M*. *hiemalis*, *T*. *harzianum* and *F*. *oxysporum*), between siderophore production and mycophagy on *R*. *solani*, between inhibition (all fungi except for *M*. *hiemalis*, *T*. *harzianum* and *F*. *oxysporum*) and purple pigmentation and between soil pH and purple pigmentation. Inhibition of all fungi except for *M*. *hiemalis*, *T*. *harzianum* and *F*. *oxysporum* was positively correlated with mycophagy on both fungi with a p value close to significance (p = 0.052 and p = 0.053, respectively for mycophagy on *M*. *hiemalis and R*. *solani*). Significant negative correlations (p ≤ 0.05) were seen between swarming and siderophore production, between swarming and fungal inhibition (all fungi except for *M*. *hiemalis*, *T*. *harzianum* and *F*. *oxysporum*), between swarming and mycophagy (for *R*. *solani* only), between weathering and siderophore production, between weathering and mycophagy (for *M*. *hiemalis* only), between soil pH and mycophagy (for *R*. *solani* only) and between mycophagy (for *M*. *hiemalis* only) and purple pigmentation. Environments harboring significantly (p ≤ 0.05) less inhibitory collimonads concerning all fungi except for *M*. *hiemalis*, *T*. *harzianum* and *F*. *oxysporum* were “dune grassland”, unfertilized grassland” and “former agricultural field”. The habitats “EMF soil” and “former agricultural field” also had significantly (p ≤ 0.05) more motile bacteria, characterized by higher swarming abilities. Habitats also differed concerning their pH values, for details we refer to S1 Table.

### Phylogenetic signal

Phylogenetic signal calculations for concatenated and 16s rDNA phylogenies can be found in Table 1. Briefly, with the housekeeping gene phylogeny we found significant phylogenetic signal for purple pigmentation, *VioA*, *VioB*, collimomycin production, inhibition of all fungi except for *F*. *oxysporum* (and *M*. *hiemalis* and *T*.*harzianum* since those fungi were not inhibited at all), chitinase production, siderophore production, swarming intensity and swarming shape, habitat and soil pH. The phylogenetic signal for mycophagy on *M*. *hiemalis* was nearly significant (p = 0.066). For the 16s phylogeny, significant phylogenetic signal was found for purple pigmentation, *VioA*, *VioB*, collimomycin production, inhibition of all fungi except for *F*. *oxysporum* (and *M*. *hiemalis* and *T*.*harzianum*, see above), chitinase production, swarming intensity and swarming shape. Soil pH was nearly significant (p = 0.073). For Bloomberg’s K, Pagel’s ʎ and Fritz’ and Purvis’ D values we refer to Table 1.

**Table 1:** Trait phylogenetic signals.

a)						
Trait category	Trait	Variable type	Bloombergs K	Pagels λ	Fritz & Purvis D	P-value
Violacein production						
	Purple pigment	binary			-0,27	< 0,0001
	*VioB*	discrete		1,00		< 0,0001
	*VioA*	discrete		1,00		< 0,0001
Collimomycin genes						
	Collimomycin	binary			-0,48	< 0,0001
Fungal inhibition						
	*R*. *solani*	binary			-0,21	< 0,0001
	*P*. *ultimum*	binary			-0,30	< 0,0001
	*F*. *oxysporum*	binary			1,02	0,5010
	*F*. *culumorum*	binary			-0,31	< 0,0001
	*A*. *niger*	binary			-0,06	< 0,0001
	*P*. *exigua*	binary			-0,32	< 0,0001
Mycophagy						
	*M*. *hiemalis*	continuous	0,55			0,0660
	*R*. *solani*	continuous	0,29			0,5140
Chitinase production						
	Chitinase	binary			0,13	0,0020
Siderophore production						
	Siderophores	continuous	0,03			0,0070
Weathering						
	Weathering	continuous	0,10			0,1560
Swarming abilities						
	Intensity	discrete		0,69		0,0001
	Shape	discrete		0,63		0,0020
Environmental data						
	Species	discrete		0,75		0,0009
	Habitat	discrete		0,98		< 0,0001
	Soil pH	continuous	0,06			0,0010
b)						
Trait category	Trait	Variable type	Bloombergs K	Pagels λ	Fritz & Purvis D	P-value
Violacein production						
	Purple pigment	binary			0,39	< 0,0001
	*VioB*	discrete		0,82		< 0,0001
	*VioA*	discrete		0,85		< 0,0001
Collimomycin genes						
	Collimomycin	binary			-0,33	< 0,0001
Fungal inhibition						
	*R*. *solani*	binary			0,06	< 0,0001
	*P*. *ultimum*	binary			0,01	< 0,0001
	*F*. *oxysporum*	binary			1,24	0,7770
	*F*. *culumorum*	binary			0,00	< 0,0001
	*A*. *niger*	binary			0,25	< 0,0001
	*P*. *exigua*	binary			-0,06	< 0,0001
Mycophagy						
	*M*. *hiemalis*	continuous	0,12			0,1070
	*R*. *solani*	continuous	0,05			0,7890
Chitinase production						
	Chitinase	binary			0,28	0,0010
Siderophore production						
	Siderophores	continuous	0,00			0,1380
Weathering						
	Weathering	continuous	0,00			0,6610
Swarming abilities						
	Intensity	discrete		0,56		< 0,0001
	Shape	discrete		0,46		0,0029
Environmental data						
	Species	discrete		0,29		0,2578
	Habitat	discrete		0,25		1,0000
	Soil pH	continuous	0,00			0,0730

Signals are derived from a) a concatenated gene phylogeny (*rpoB*, *gyrB*, 16s) and b) 16s only phylogeny. Type of variable (continuous, discrete and binary) and respective phylogenetic signal measurements (Bloombergs’ K, Pagels λ and Fritz’ and Purvis’ D) as well as their significance is indicated.

## Discussion

By combining the constructed high-resolution phylogeny with a set of functional traits, we found evidence for phylogenetically conserved trait divergence. As indicated by significant phylogenetic signal, several traits potentially involved in interactions between collimonads and fungi, are not randomly distributed but tend to group with phylogeny (Table 1, Fig 2). These traits are the inhibition of a diverse set of fungi, namely *R*. *solani*, *P*. *ultimum*, *F*. *culmorum*, *A*. *niger* and *P*. *exigua*, the production of violacein and collimomycin, mycophagous growth on *M*. *hiemalis*, the production of chitinases, swarming ability and swarming shape.

The aforementioned traits do not (or to a lesser extent) carry phylogenetic signal when being examined on basis of a 16s rDNA phylogeny only. This indicates that with a lower taxonomic resolution, the phylogenetic signal gets “diluted” and it gets harder to assign specific traits to clusters of phylogenetically related bacteria. It has been shown that many genetically complex traits are phylogenetically not widely distributed but rather conserved and deeply rooted in the phylogenetic tree, indicating that those traits might not be associated with very fine-scale diversity [12, 39]. Compared to Martiny, Treseder [12], spanning the whole kingdom of *Bacteria* (and also *Archeae*), we assessed very fine-level trait dispersal in the bacterial genus *Collimonas*. Our study confirms the suggestion by Martiny, Treseder [12] that fine-resolution trait dispersal and phylogeny are required to obtain detailed information on phylogenetic signal at fine taxonomic levels.

Despite the fact that we find related strains to possess similar traits (phylogenetic signal) we observe that the strength of the phylogenetic signal (dispersal over the phylogenetic tree) differs (Table 1, Fig 2). Traits like violacein and chitinase production are deeply rooted in the tree, whereas fungal inhibition is not. An explanation for this could be that although violacein has antifungal activity, its primary function is not that of a diffusible fungal inhibitor. It rather evolved at an evolutionary earlier time point, probably before the development of mycophagy and could have served in the inhibition of bacterivorous predators. Violacein is produced and stored intracellularly and for *Janthinobacterium* bacteria it has been shown to be toxic to bacterivorous nematodes, upon ingestion *[40]*). Furthermore, the compound has not been proven to be actively used as an antifungal compound by collimonads. Indeed, our study reveals that many *Collimonas* strains that were not scored positive for violacein production had similar antifungal inhibition patterns as violacein-producing strains (Fig 2). Hence, antifungal activity of violacein containing *Collimonas* bacteria in *in vitro* assays is probably due to other (diffusible) secondary metabolites. The same could hold for the acquisition of chitinase genes, which might have taken place at an evolutionary earlier time point, the original purpose being degradation of chitin present in various sources like invertebrate exoskeletons, decaying fungal remainders etc. [41]. The closely related genus *Janthinobacterium* harbors very efficient degraders of chitin [42]. However, *Collimonas* bacteria are poor degraders of crystallized chitin. Therefore, they may have lost accompanying enzymes that are needed to degrade crystallized and cross-polymerized chitin and use their chitinases especially in attack of hyphal tips of fungi, where chitin polymers are in their native form and are most vulnerable for lytic enzymes [3, 41].

The observed differences in the depth of rooting and the dispersal of the traits on the phylogenetic tree (phylogenetic signal) do not indicate the existence of distinct *Collimonas* “ecotypes” that could possibly be grouped by functional traits only (Fig 2). A trait like fungal inhibition could have evolved several times in different *Collimonas* strains, followed by further investment in strain specific traits. Other traits like purple pigmentation for example could be relicts from the sister genus *Janthinobacterium*. Those traits might have provided benefits to some collimonads and were therefore partially maintained (how far those traits could still contribute to the formation of possible ecotypes is, however, unclear). It would be necessary to sequence violacein genes of known violacein producing *Collimonas* sister genera in order to trace the evolution of violacein production in the *Oxalobacteraceae*. Interestingly, we did not find evidence for nitrogenase genes (*nifH*) in collimonads. As opposed to the results for violacein, these genes seem to have gotten lost upon adaptation to the mycophagous lifestyle.

The distribution of functional traits indicates potential trade-offs between mineral weathering and swarming as well as between fungal inhibition, mycophagy and siderophore production (Fig 3).

Indications for a possible trade-off between swarming and siderophore production has been shown before by Cheng, de Bruijn [43]. These authors showed that the *Pseudomonas fluorescens* SBW25 *gacS* mutant, impaired in swarming, produced a higher amount of siderophores than the corresponding wild type and *vice versa*. Tremblay and Deziel [44] show that actively swarming cells of *Pseudomonas aeruginosa* have generally down-regulated iron acquisition genes as compared to cells in the center of the colony that do not swarm. Also for collimonads, this trade-off would make sense. Siderophores are molecules which are most useful when being produced at high local concentration. Their production requires energy which would be optimally invested when combined with low bacterial movement.

We also found negative correlations (possible trade-offs) between swarming and mycophagy and swarming and fungal inhibition (Fig 3). Like for the production of siderophores, the production of antifungal compounds, a pre-requisite for both, would only pay off when they would be produced locally and in high concentrations. Collimonads may, however use motility in order to locate a potential host fungus and produce antifungal metabolites, once the host is located. Motility and chemotaxis have already been indicated to be important traits of collimonads [45, 46].

Our study also indicated biogeography effects, meaning that certain *Collimonas* genotypes are more prone to be found in certain habitat types than in others. Unfertilized grasslands, abandoned agricultural fields and dune grasslands appear to contain different collimonads than arctic habitats, forests, river dunes e.g. (Fig 3, S1 Table). This differentiation is only linked with two traits, namely less motility in terms of swarming intensity and less fungal inhibition for strains isolated from “former agricultural fields”. Therefore, it may indicate that collimonads interact with fungi in a different way or with other fungal hosts, depending on the habitat. We don’t know, however, whether the presence or absence of fungal hosts, the competition with other bacteria or the abiotic conditions, traits and factors that we did not measure, further influence the distribution of collimonads. We also found differences between the soil pH of different *Collimonas* habitats (Fig 3). We thus cannot exclude a possible pH effect on the distribution of *Collimonas* strains.

In this study, we used different methods to measure phylogenetic signal, depending on the nature of the trait. For Pagels ʎ and Fritz’ and Purvis’ D, used for discrete and binary trait data, respectively, the measured values for phylogenetic signal were well in the range of values detected in other studies [10]. While still being significant, Bloomberg’s K values were below 1, by definition indicating less trait evolution than expected under Brownian motion. There is another study that reports the same extraordinarily low but significantly different Bloomberg’s K values for bacteria. Here, the authors found that pH, an environmental parameter that is known to profoundly shape bacterial lifestyle [47, 48], shows phylogenetic signal for methanotrophic bacteria in a similar range as we measured in our study [49]. It remains to be seen if this tendency is common for the evolution of microbial traits.

It is important to note that the observed changes in trait investment represent static data, based on the presence and absence of genes or on the conduction of phenotypic assays under specific conditions. There are two reasons why the results of such studies should be carefully interpreted: a) It has been shown that traits can get lost upon cultivation. Eydallin, Ryall [50] could for example show that *E*. *coli* strains experienced changes in morphotype, metabolism and fitness after a few days of cultivation, b) We did not assess the adaptive potential of *Collimonas* strains. It is, however, highly probable that the expression of bacterial functional traits depends on the abiotic and biotic context. It has for example been shown in previous experiments with collimonads that movement and swarming is dependent on the availability of fungal signal compounds, having an inversely proportional effect on bacterial movement [51]. We chose to conduct all assays at 20°C. This had practical reasons: All collimonads and interacting fungi grew very slow at 4°C, even though some collimonads were isolated from arctic habitats. There is, however, the possibility that these artificially high temperatures might have affected phenotypic assays of the artic *Collimonas* isolates.

One of the main drivers of the *Collimonas* grouping is the ability to inhibit fungi, to feed on them and to produce antimicrobial compounds like collimomycin. Production of antifungal compounds and inhibition of fungi is highly dependent on culture-conditions [14, 51] and collimonads cultured in liquid have shown to express antifungal traits to a lesser extent compared to cultures on solid medium. We pre-cultured collimonads on solid, rather than liquid medium. This has been shown to trigger adaptations in *E*.*coli* and might explain why the fungal inhibition results for specific strains, e.g. Ter331, obtained in this study differ from previous ones [52]. Although we only found a trend and not a significant positive relationship between fungal inhibition and mycophagy, this result gives some support to earlier suggestions that antifungal compounds are an important factor for the mycophagous growth of collimonads [3]. Our study however indicates that fungal inhibition carries phylogenetic signal, whereas mycophagy does not. Thus, suggesting that many different factors might contribute to mycophagy, inhibitory compounds probably being only a part of this.

To summarize, our study shows that MLSA enables a more detailed description of phylogenetic relationships within the genus *Collimonas* than 16S rDNA sequences. Using this multi-gene, high-resolution phylogeny we show that a set of traits that are possibly involved in interaction of collimonads and their host fungi are phylogenetically conserved. We find indications for trade-offs for *Collimonas’* trait possession that might be a pre-requisite for the colonization of certain habitats.

## Supporting Information

S1 Table Overview on trait data, habitat data, species affiliation and reference. "Strain" indicates the name of the *Collimonas* isolate. Collimomycin genes (cluster K, see Fritsche et al 2014) were either not determined (NA), not present (1) or present (2). Mineral weathering (free Fe released) was either quantified in mgL^-1^, see Uroz et al 2009) or not determined (NA). Siderophores were quantified by a CAS-overlay assay (mm diameter of colony sourrounding halo), NA indicates not determined. Chitinase activity was quantified by halo formation on Chitin agar, no degradation (0) and degradation (1) are indicated. In fungal inhibition assays (*R*. *solani*, *M*. *hiemalis*, *P*. *ultimum*, *F*. *oxysporum*, *F*. *culmorum*, *A*.*niger*, *P*. *exigua* and *T*. *harzianum*, “1” indicates inhibition of the respective fungus and “0” indicates no inhibition of the respective fungus. Purple = “1” indicates a purple pigmentation of the respective strain, purple = “0” no purple pigmentation. *VioA*/B = “0” means that the respective genes, *VioA* and *VioB*, could not be amplified (Hakvåg, et al. 2009), *VioA*/B = “1” means that fragments could be amplified but they do not have the right size, *VioA*/B = 2 means that right size fragments were amplified. Mycophagy ratios were determined for possible growth on *M*. *hiemalis* and *T*. *harzianum* (NA indicates no meassurement and “0” indicates that the data has been deleted from the dataset because of too much variation between replicates. Soil pH indicates the pH of the soil from which the respective strain was isolated (NA = not determined). Country stands for the country of isolation (“1” = The Netherlands, “2” = France, “3” = Finnland). Species is either not determined (NA), *C*. *fungivorans* (1), *C*. *arenae* (2) or *C*. *pratensis* (3). Habitat indicates the habitat from which the strain was isolated, either being “NA” = no data available, “emf_soil” = hypha of ectomycorrhizal fungus, “arctic_bulk” = arctic bulk soil, “arctic_forest” = arctic forest soil, “arctic_tundra” = arctic tundra soil, “endophyte” = the inside of a plant root, “former_agr_field” = abandonned agricultural field site, “beech_forest” = beech forest soil, “unfert_grassland” = unfertilized grassland soil, “coastal_outer_dunes” = coastal outer dune soil, “marsh” = marshland soil, “riverdune_grassland” = riverdune grassland soil, “heathland” = heathland soil, “dune_grassland” = dune grassland soil. Swarming behaviour was characterized in terms of intensity (“0” = no swarming, “1” = low, “2” = moderate, “3” = strong) and shape. Shapes were variable and descriptive names were given. Reference indicates the reference for the isolation of the respective strains.
(XLSX)

S2 Table Overview on statistical methods and data. Abbreveations: sqrt = sqare root transformation of the response variable, df = degrees of freedom, p = p-value, F = F-value, t = t-value, leveneTest = result of Levenes Test, sum sq = sum of squares, means sq = mean sum of squares, diff indicates the difference in means in Tukeys HSD, lwr and upr confidence intervals in Tukeys HSD test, z = z-value, muc_ratio = Mycophagy ratio *M*. *hiemalis*, rhiz_ratio = Mycophagy ratio *R*. *solani*. Fungal inhibition abbreviations: Pult = *P*. *ultimum*, Anig = *A*. *niger*, Rsol = *R*. *solani*, Mhie = *M*. *hiemalis*, Foxy = *F*. *oxysporum*, Fcul = *F*. *culmorum*, Phoma = *P*. *exigua*, Thar = *T*. *harzianum*. collimomycin = collimomycin genes (cluster K), weathering = freed iron through mineral weathering, siderophores = siderophore production on CAS medium, swarming = swarming intensity, purple = purple pigmentation, Habitat describes the habitat of isolation and is either “no_data” = no data available, “emf_soil” = hypha of ectomycorrhizal fungus, “arctic_bulk” = arctic bulk soil, “arctic_forest” = arctic forest soil, “arctic_tundra” = arctic tundra soil, “endophyte” = the inside of a plant root, “former_agr_field” = abandonned agricultural field site, “beech_forest” = beech forest soil, “unfert_grassland” = unfertilized grassland soil, “coastal_outer_dunes” = coastal outer dune soil, “marsh” = marshland soil, “riverdune_grassland” = riverdune grassland soil, “heathland” = heathland soil or “dune_grassland” = dune grassland soil. Correlations between traits were either tested with a) linear models, b) Pearsons correlation, c) t-test d) Pearsons chi-squared test e) Analysis of Variance, followed by posthoc tests for treatment level differences (Tukeys Honest Significant Difference (HSD)) or f) logistic regression models.
(XLSX)

S3 Table Primer that were developed for this study. Amplified gene, respective sequences of forward and reverse primers, annealing temperatures, and resulting amplicon sizes are given.
(XLS)

## References

[1] Hibbing ME, Fuqua C, Parsek MR, Peterson SB. Bacterial competition: surviving and thriving in the microbial jungle. Nat Rev Microbiol. 2010;8(1):15–25. 10.1038/Nrmicro2259 . 19946288 PMC2879262

[2] Folse HJ, Allison SD. Cooperation, competition, and coalitions in enzyme-producing microbes: social evolution and nutrient depolymerization rates. Frontiers in Microbiology. 2012;3:1–10. Artn 338 10.3389/Fmicb.2012.00338 . 23060866 PMC3459022

[3] Leveau JHJ, Uroz S, de Boer W. The bacterial genus *Collimonas*: mycophagy, weathering and other adaptive solutions to life in oligotrophic soil environments. Environ Microbiol. 2010;12(2):281–92. 10.1111/j.1462-2920.2009.02010.x . 19638176

[4] Mela F, Fritsche K, de Boer W, van Veen JA, de Graaff LH, van den Berg M, et al Dual transcriptional profiling of a bacterial/fungal confrontation: *Collimonas fungivorans* versus *Aspergillus niger*. Isme Journal. 2011;5(9):1494–504. 10.1038/ismej.2011.29 . 21614084 PMC3160687

[5] Mela F, Fritsche K, de Boer W, van den Berg M, van Veen JA, Maharaj NN, et al Comparative genomics of bacteria from the genus *Collimonas*: linking (dis)similarities in gene content to phenotypic variation and conservation. Environmental Microbiology Reports. 2012:no-no. 10.1111/j.1758-2229.2012.00336.x 23760828

[6] Hoppener-Ogawa S, Leveau JHJ, Hundscheid MPJ, van Veen JA, de Boer W. Impact of *Collimonas* bacteria on community composition of soil fungi. Environ Microbiol. 2009;11(6):1444–52. 10.1111/j.1462-2920.2009.01872.x . 19260938

[7] Rudnick MB, van Veen JA, de Boer W. Baiting of rhizosphere bacteria with hyphae of common soil fungi reveals a diverse group of potentially mycophagous secondary consumers. Soil Biol Biochem. 2015;88(0):73–82. 10.1016/j.soilbio.2015.04.015.

[8] Hoppener-Ogawa S, Leveau JHJ, van Veen JA, De Boer W. Mycophagous growth of *Collimonas* bacteria in natural soils, impact on fungal biomass turnover and interactions with mycophagous *Trichoderma* fungi. Isme Journal. 2009;3(2):190–8. 10.1038/ismej.2008.97 . 18923455

[9] Deveau A, Palin B, Delaruelle C, Peter M, Kohler A, Pierrat JC, et al The mycorrhiza helper *Pseudomonas fluorescens* BBc6R8 has a specific priming effect on the growth, morphology and gene expression of the ectomycorrhizal fungus *Laccaria bicolor* S238N. New Phytol. 2007;175(4):743–55. 10.1111/j.1469-8137.2007.02148.x . 17688589

[10] Münkemüller T, Lavergne S, Bzeznik B, Dray S, Jombart T, Schiffers K, et al How to measure and test phylogenetic signal. Methods in Ecology and Evolution. 2012;3(4):743–56. 10.1111/j.2041-210X.2012.00196.x .

[11] Diaz S, Purvis A, Cornelissen JHC, Mace GM, Donoghue MJ, Ewers RM, et al Functional traits, the phylogeny of function, and ecosystem service vulnerability. Ecology and Evolution. 2013;3(9):2958–75. 10.1002/Ece3.601 . 24101986 PMC3790543

[12] Martiny AC, Treseder K, Pusch G. Phylogenetic conservatism of functional traits in microorganisms. Isme Journal. 2013;7(4):830–8. 10.1038/ismej.2012.160 . 23235290 PMC3603392

[13] Uroz S, Calvaruso C, Turpault MP, Sarniguet A, de Boer W, Leveau JHJ, et al Efficient mineral weathering is a distinctive functional trait of the bacterial genus Collimonas. Soil Biol Biochem. 2009;41(10):2178–86. 10.1016/j.soilbio.2009.07.031 .

[14] Fritsche K, van den Berg M, de Boer W, van Beek TA, Raaijmakers JM, van Veen JA, et al Biosynthetic genes and activity spectrum of antifungal polyynes from *Collimonas fungivorans* Ter331. Environ Microbiol. 2014;16(5):1334–45. 10.1111/1462-2920.12440 . 24588891

[15] de Rooij-Van der Goes PCEM, Van de Putten WH, Van Dijk C. Analysis of nematodes and soil-borne fungi from *Ammophila arenaria* (Marram Grass) in dutch coastal foredunes by multivariate techniques. Eur J Plant Pathol. 1995;101(2):149–62. .

[16] Sutherland IW, Kennedy L. Polysaccharide lyases from gellan-producing *Sphingomonas spp*. Microbiology. 1996;142:867–72. . 8936312 10.1099/00221287-142-4-867

[17] Xavier JB, Kim W, Foster KR. A molecular mechanism that stabilizes cooperative secretions in *Pseudomonas aeruginosa*. Mol Microbiol. 2011;79(1):166–79. 10.1111/j.1365-2958.2010.07436.x . 21166901 PMC3038674

[18] Perez-Miranda S, Cabirol N, George-Tellez R, Zamudio-Rivera LS, Fernandez FJ. O-CAS, a fast and universal method for siderophore detection. J Microbiol Methods. 2007 7. 10.1016/j.mimet.2007.03.023 17507108

[19] Schneider CA, Rasband WS, Eliceiri KW. NIH Image to ImageJ: 25 years of image analysis. Nat Methods. 2012;9(7):671–5. . 22930834 10.1038/nmeth.2089 PMC5554542

[20] De Boer W, Klein Gunnewiek PJK, Kowalchuk GA, Van Veen JA. Growth of chitinolytic dune soil beta-subclass Proteobacteria in response to invading fungal hyphae. Appl Environ Microbiol. 2001;67(8):3358–62. 10.1128/AEM.67.8.3358-3362.2001 . 11472904 PMC93028

[21] Maiden MCJ. Multilocus sequence typing of bacteria. Annu Rev Microbiol. 2006;60:561–88. 10.1146/annurev.micro.59.030804.121325 . 16774461

[22] Walters WA, Caporaso JG, Lauber CL, Berg-Lyons D, Fierer N, Knight R. PrimerProspector: de novo design and taxonomic analysis of barcoded PCR primers. Bioinformatics. 2011 10.1093/bioinformatics/btr087 PMC3072552 21349862

[23] Moore E, Arnscheidt A, Krüger A, Strömpl C, Mau M. Simplified protocols for the preparation of genomic DNA from bacterial cultures Kowalchuk GA dBF, Head IM, Akkermans ADL, van Elsas JD, editor. Dordrecht: Kluwer Academic Publishers; 2004.

[24] Weisburg WG, Barns SM, Pelletier DA, Lane DJ. 16s Ribosomal DNA Amplification for Phylogenetic Study. J Bacteriol. 1991;173(2):697–703. . 1987160 10.1128/jb.173.2.697-703.1991 PMC207061

[25] Ando S, Goto M, Meunchang S, Thongra-ar P, Fujiwara T, Hayashi H, et al Detection of nifH Sequences in sugarcane (Saccharum officinarum L.) and pineapple (Ananas comosus [L.] Merr.). Soil Sci Plant Nutr. 2005;51(2):303–8. 10.1111/j.1747-0765.2005.tb00034.x .

[26] Hakvåg S, Fjærvik E, Klinkenberg G, Borgos SE, Josefsen K, Ellingsen T, et al Violacein-Producing Collimonas sp. from the Sea Surface Microlayer of Costal Waters in Trøndelag, Norway. Mar Drugs. 2009;7(4):576–88. 10.3390/md7040576 20098599 PMC2810235

[27] Hall TA. BioEdit: a user-friendly biological sequence alignment editor and analysis program for Windows 95/98/NT. Nucl Acids Symp Ser. 1999;41:95–8.

[28] Tamura K, Peterson D, Peterson N, Stecher G, Nei M, Kumar S. MEGA5: molecular evolutionary genetics analysis using maximum likelihood, evolutionary distance, and maximum parsimony methods. Mol Biol Evol. 2011;28(10):2731–9. 10.1093/molbev/msr121 21546353 PMC3203626

[29] Xia X, Xie Z. DAMBE: Software Package for Data Analysis in Molecular Biology and Evolution. J Hered. 2001;92(4):371–3. 10.1093/jhered/92.4.371 11535656

[30] Fritz SA, Purvis A. Selectivity in Mammalian Extinction Risk and Threat Types: a New Measure of Phylogenetic Signal Strength in Binary Traits. Conserv Biol. 2010;24(4):1042–51. 10.1111/j.1523-1739.2010.01455.x . 20184650

[31] Blomberg SP, Garland T, Ives AR. Testing for phylogenetic signal in comparative data: Behavioral traits are more labile. Evolution. 2003;57(4):717–45. 10.1111/j.0014-3820.2003.tb00285.x . 12778543

[32] Pagel M. Inferring the historical patterns of biological evolution. Nature. 1999;401(6756):877–84. 10553904 10.1038/44766

[33] Uroz S, Calvaruso C, Turpaul MP, Pierrat JC, Mustin C, Frey-Klett P. Effect of the mycorrhizosphere on the genotypic and metabolic diversity of the bacterial communities involved in mineral weathering in a forest soil. Appl Environ Microbiol. 2007;73(9):3019–27. 10.1128/Aem.00121-07 . 17351101 PMC1892860

[34] Hoppener-Ogawa S, Leveau JHJ, Smant W, van Veen JA, de Boer W. Specific detection and real-time PCR quantification of potentially mycophagous bacteria belonging to the genus *Collimonas* in different soil ecosystems. Appl Environ Microbiol. 2007;73(13):4191–7. 10.1128/Aem.00387-07 . 17483278 PMC1932782

[35] Mannisto MK, Haggblom MM. Characterization of psychrotolerant heterotrophic bacteria from Finnish Lapland. Syst Appl Microbiol. 2006;29(3):229–43. 10.1016/j.syapm.2005.09.001 . 16564959

[36] Nissinen RM, Mannisto MK, van Elsas JD. Endophytic bacterial communities in three arctic plants from low arctic fell tundra are cold-adapted and host-plant specific. FEMS Microbiol Ecol. 2012;82(2):510–22. 10.1111/j.1574-6941.2012.01464.x . 22861658

[37] de Boer W, Leveau JHJ, Kowalchuk GA, Gunnewiek PJAK, Abeln ECA, Figge MJ, et al Collimonas fungivorans gen. nov., sp nov., a chitinolytic soil bacterium with the ability to grow on living fungal hyphae. Int J Syst Evol Microbiol. 2004;54:857–64. 10.1099/ijs.0.02920-0 . 15143036

[38] Hoppener-Ogawa S, de Boer W, Leveau JHJ, van Veen JA, de Brandt E, Vanlaere E, et al *Collimonas arenae* sp nov and *Collimonas pratensis* sp nov., isolated from (semi-)natural grassland soils. Int J Syst Evol Microbiol. 2008;58:414–9. 10.1099/ijs.0.65375-0 . 18218941

[39] Martiny JB, Jones SE, Lennon JT, Martiny AC. Microbiomes in light of traits: A phylogenetic perspective. Science. 2015;350(6261):aac9323 10.1126/science.aac9323 . 26542581

[40] Hornung C, Poehlein A, Haack FS, Schmidt M, Dierking K, Pohlen A, et al The *Janthinobacterium* sp HH01 genome encodes a homologue of the *V*. *cholerae* CqsA and *L*. *pneumophila* LqsA autoinducer synthases. PLoS ONE. 2013;8(2). ARTN e55045 10.1371/journal.pone.0055045 . PMC3566124 23405110

[41] Bai Y, Eijsink VGH, Kielak AM, van Veen JA, de Boer W. Genomic comparison of chitinolytic enzyme systems from terrestrial and aquatic bacteria. Environ Microbiol. 2014 10.1111/1462-2920.12545 24947206

[42] Kielak AM, Cretoiu MS, Semenov AV, Sorensen SJ, van Elsas JD. Bacterial chitinolytic communities respond to chitin and pH alteration in soil. Appl Environ Microbiol. 2013;79(1):263–72. 10.1128/Aem.02546-12 . 23104407 PMC3536121

[43] Cheng X, de Bruijn I, van der Voort M, Loper JE, Raaijmakers JM. The Gac regulon of *Pseudomonas fluorescens* SBW25. Environmental Microbiology Reports. 2013;5(4):608–19. 10.1111/1758-2229.12061 23864577

[44] Tremblay J, Deziel E. Gene expression in *Pseudomonas aeruginosa* swarming motility. BMC Genomics. 2010;11 Artn 587 10.1186/1471-2164-11-587 . PMC3091734 20961425

[45] Rudnick MB, van Veen JA, de Boer W. Oxalic acid: a signal molecule for fungus-feeding bacteria of the genus *Collimonas*? Environmental Microbiology Reports. 2015:n/a-n/a. 10.1111/1758-2229.12290 25858310

[46] Wolf AB, Rudnick M-B, de Boer W, Kowalchuk GA. Early colonizers of unoccupied habitats represent a minority of the soil bacterial community2015 2015-03-16 00:00:00. 10.1093/femsec/fiv024 25778508

[47] Lauber CL, Strickland MS, Bradford MA, Fierer N. The influence of soil properties on the structure of bacterial and fungal communities across land-use types. Soil Biol Biochem. 2008;40(9):2407–15. 10.1016/j.soilbio.2008.05.021.

[48] Sharp CE, Brady AL, Sharp GH, Grasby SE, Stott MB, Dunfield PF. Humboldt/'s spa: microbial diversity is controlled by temperature in geothermal environments. ISME J. 2014;8(6):1166–74. 10.1038/ismej.2013.237 24430481 PMC4030231

[49] Krause S, van Bodegom PM, Cornwell WK, Bodelier PLE. Weak phylogenetic signal in physiological traits of methane-oxidizing bacteria. J Evol Biol. 2014;27(6):1240–7. 10.1111/jeb.12401 24797710

[50] Eydallin G, Ryall B, Maharjan R, Ferenci T. The nature of laboratory domestication changes in freshly isolated *Escherichia coli* strains. Environ Microbiol. 2014;16(3):813–28. 10.1111/1462-2920.12208 . 23889812

[51] Rudnick MB, van Veen JA, de Boer W. Oxalic acid: a signal molecule for fungus-feeding bacteria of the genus *Collimonas*? Environmental Microbiology Reports. 2015 10.1111/1758-2229.12290 25858310

[52] De Boer W, Gunnewiek PJAK, Lafeber P, Janse JD, Spit BE, Woldendorp JW. Anti-fungal properties of chitinolytic dune soil bacteria. Soil Biol Biochem. 1998;30(2):193–203. .

